# Multi-factor orthogonal optimization and experimental performance study of flow passages in a vertical mixed-flow pump unit

**DOI:** 10.1371/journal.pone.0343448

**Published:** 2026-06-08

**Authors:** Jiamin Zhang, Zhuangzhuang Sun, Songshan Chen, Ning Lü, Yujing Qiao

**Affiliations:** 1 School of Mechanical Engineering, Yangzhou Polytechnic University, Yangzhou, China; 2 College of Electrical, Energy and Power Engineering, Yangzhou University, Yangzhou, China; NED University of Engineering and Technology, PAKISTAN

## Abstract

Flow passage optimization is an essential approach for enhancing the efficiency and operational stability of vertical mixed-flow pump units. To address the limitations of the traditional single-factor control variable method—which ignores parameter interaction effects and relies on empirical judgment for scheme screening, leading to low optimization efficiency and insufficient engineering adaptability—this study proposes a multi-factor interactive flow passage optimization methodology that deeply couples orthogonal experimental design with Computational Fluid Dynamics (CFD) simulations. A multi-indicator quantitative evaluation system encompassing “hydraulic loss, velocity uniformity, and weighted average angle” was constructed. Taking a large-scale drainage pumping station as the research object, key parameter combinations were systematically covered through orthogonal experiments. The optimal intake flow passage scheme was screened via CFD simulation, which was verified to have a hydraulic loss of only 0.104 m, an outlet velocity uniformity of 97.06%, and a weighted average angle of 84.82°, approaching the ideal vertical inflow, thereby effectively reducing flow impact losses. The optimal discharge flow passage scheme demonstrated smooth flow patterns without significant flow separation and was fully compatible with the spatial layout of the pumping station. Model test validation showed that under the design head condition of 7.1 m, the pump unit efficiency reached 77.34% with a flow rate of 11.38 m^3^/s. The error between CFD simulation and experimental results was less than 5%, meeting the design requirements. This study provides a scientifically efficient and engineeringly feasible technical pathway for flow passage optimization in similar vertical mixed-flow pump units.

## 1. Introduction

Vertical mixed-flow pumps, leveraging their performance advantages of wide head range and large flow capacity, have become core equipment in major water conservancy projects such as inter-basin water transfer, urban flood drainage, and agricultural irrigation and drainage [1–3]. As critical components of the pump unit’s flow passage system, the intake and discharge flow passages directly determine the energy transfer efficiency from the sump to the impeller chamber and from the guide vane body to the outlet sump [4,5]. The intake flow passage is required to achieve smooth flow acceleration and turning, and its flow uniformity directly affects the pump’s suction conditions. The discharge flow passage, on the other hand, undertakes the functions of kinetic energy recovery and flow rectification, with the rationality of its profile playing a decisive role in flow capacity and hydraulic loss [6–8]. Therefore, conducting optimization design for intake and discharge flow passages holds significant engineering importance for enhancing the overall performance of pump units.

In recent years, Computational Fluid Dynamics (CFD) technology and model test methods have been widely applied in the optimization of hydraulic machinery [9–11]. Scholars both domestically and internationally have carried out extensive research on pump unit flow passage optimization, forming a synergistic technical approach of numerical simulation and model test validation [12–14]. In terms of numerical simulation, research has focused on performance comparison and geometric optimization of flow passages [15–22]. Zhang et al. [6] combined numerical and experimental methods to improve the hydraulic performance of an existing pumping station through impeller and diffuser redesign, analyzing factors like flow distribution and loss. Wang et al. [7] specifically focused on the optimization design of inlet and outlet passages for tank-type axial-flow pumping stations. Ji et al. [17] systematically reviewed the internal flow analysis and optimization methods for centrifugal pumps, including discussions on turbulence model selection and numerical strategies that have enhanced the accuracy of flow calculations. Furthermore, Wu et al. [20] conducted a detailed numerical and experimental investigation of the flow in a 30° inclined intake channel and its effect on pump performance. Constantinescu and Patel [22] developed a numerical model for the simulation of pump-intake flow and vortices, contributing to the analysis of complex inflow conditions. Regarding model testing and experimental validation, extensive work has been conducted to provide empirical evidence and refine design standards [23–28]. Tan et al. [24] investigated the hydraulic characteristics of a box culvert discharge passage in a low-head pumping station through combined experiments and numerical simulation. Paik et al. [8] experimentally investigated the critical flow patterns in a pump sump and their direct effects on pump performance. Gonzalez et al. [29] studied the steady and unsteady radial forces in a centrifugal pump through experimental measurements, linking geometry to dynamic loads. Yang et al. [11] analyzed the effects of geometric parameters on performance and pressure pulsations through experimental studies. Research on scale effects and advanced measurement techniques is also vital. Padmanabhan and Hecker [30] addressed the fundamental issue of scale effects in pump sump models through experimental investigation [31–33]. Chu et al. [31] utilized advanced experimental methods to explore the relationship between unsteady flow, pressure fluctuations, and noise in a centrifugal pump. Finally, Lu et al. [33] employed combined model tests and numerical simulations to study the complex flow in a forebay with asymmetric inflows, providing valuable data for validating numerical models under realistic engineering conditions.

The aforementioned research outcomes have provided important theoretical and practical foundations for understanding the factors influencing the overall hydraulic performance of pump units and for improving the overall efficiency of pumping stations [34–35]. However, significant limitations persist. Existing optimization approaches predominantly employ the single-factor control variable method, which neglects the interaction effects among multiple parameters. This leads to a strong element of blindness in scheme screening, an inability to quantify the influence weight of each parameter on hydraulic performance, and consequently, low optimization efficiency [36–38].

To address this core issue, this study focuses on the vertical mixed-flow pump unit and proposes an intake flow passage optimization methodology that deeply couples orthogonal experimental design with CFD numerical simulation, thereby overcoming the limitations of traditional single-factor optimization. By scientifically arranging test schemes using an L9(3^3^) orthogonal array, only 9 simulation sets are required to comprehensively cover full-combination scenarios of key intake flow passage parameters—namely throat height, bend section length, and transition arc length. This approach significantly reduces computational costs while accurately capturing parameter interactions and their primary/secondary influence weights. Subsequently, the optimal flow passage combination is screened based on key indicators such as hydraulic loss and velocity uniformity. An entropy production distribution comparison is conducted among four high-performing flow passage schemes to further verify the flow field advantages of the optimal scheme. Furthermore, a full three-dimensional model encompassing the forebay, elbow-type intake flow passage, impeller, guide vanes, and straight pipe-type discharge flow passage is constructed for overall pump unit performance simulation and optimization, thereby verifying the reliability of the intake flow passage optimization method. Finally, a high-precision model test rig is established to conduct energy characteristic tests. A comparison between the experimental results and CFD simulation data validates the rationality and engineering adaptability of the optimized scheme. The research findings can provide theoretical basis and practical reference for the flow passage optimization design of similar vertical mixed-flow pump units.

## 2. Theoretical principles of hydraulic performance indicators for pumping stations

The actual hydraulic loss of a pumping station is a core metric for evaluating the energy transfer efficiency of its flow passages [39]. A smaller value indicates higher energy conversion efficiency within the passages. In practical engineering design, flow passage performance is influenced by the interaction of multiple parameters. This study establishes a multi-parameter weighting model based on the Bernoulli equation and entropy production theory to rapidly obtain optimization results.

### 2.1 Basic parameters of the pumping station and pump unit

**Fig 1:**
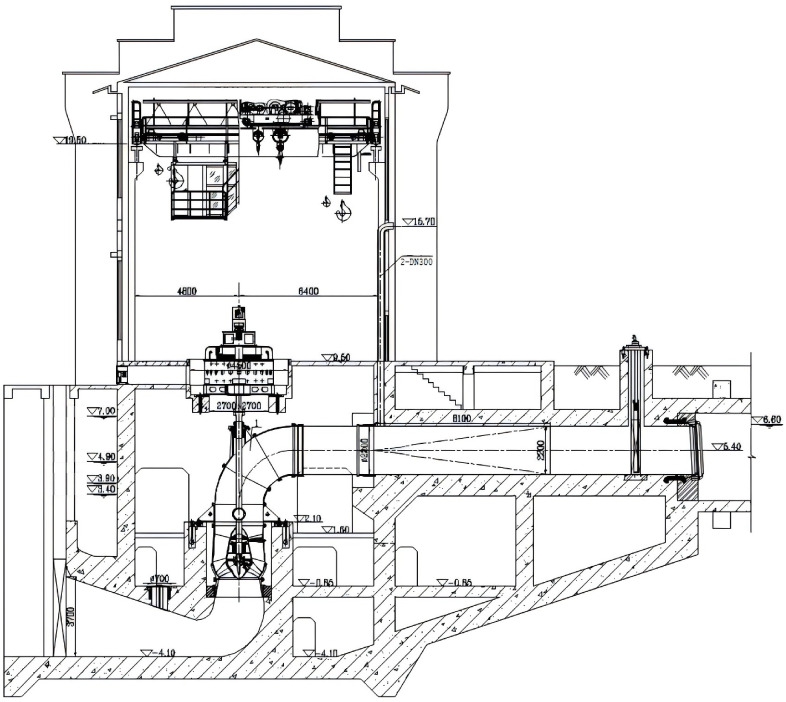
Longitudinal section view of the pumping station.

The longitudinal profile of the actual pumping station project is shown in Fig 1, and its key design parameters are listed in Table 1. The pump unit employs the TJ11-HL-02 hydraulic model. The prototype impeller diameter is *D* = 1900 mm, and the rotational speed is *n* = 214.3 r/min.

**Table 1:** Design characteristic parameters of a certain pumping station.

Category	Value
Design Flow Rate	≥10.6m^3^/s
Characteristic Water Level	3.9m
Sump Minimum Operating Level	3.4m
Sump Maximum Operating Level	4.9m
Outlet Sump Design Operating Level	11.0m
Outlet Sump Minimum Operating Level	6.6m
Outlet Sump Maximum Operating Level	12.7m
Characteristic Head	7.1m
Average Net Head	2.7m
Minimum Net Head	1.7m
Maximum Net Head	8.8m
Unit Parameter: Installation Elevation	▽-0.55m
Intake Flow Passage Invert Elevation	▽-4.10m
Intake Flow Passage Width	5.80m

### 2.2 Hydraulic performance evaluation indicators

The primary function of the intake flow passage is to guide water smoothly and uniformly from the forebay or sump to the pump impeller inlet. The discharge flow passage, through a gradually expanding cross-section, reduces the flow velocity progressively, converting part of the kinetic energy into pressure energy [40]. To improve the hydraulic efficiency of the pump unit, both should exhibit minimal hydraulic loss. The hydraulic loss for the intake and discharge flow passages is calculated based on the Bernoulli equation and is defined as:


$$\begin{array}{c} \Delta h=E_{\text{1}}-E_{\text{2}}=\left(\frac{P_{\text{1}}}{\rho g}-\frac{P_{\text{2}}}{\rho g}+Z_{\text{1}}-Z_{\text{2}}\right)+\left(\frac{u\text{12}-u\text{22}}{\text{2}g}-\frac{u\text{12}-u\text{22}}{\text{2}g}\right) \end{array}$$ (1)

where: *E*_1_ is the total energy at the inlet section of the flow passage; *E*_*2*_ is the total energy at the outlet section of the flow passage; *P*_1_ and *P*_2_ are the static pressure at various points of the pump unit’s inlet and outlet cross-sections, respectively; g is the acceleration due to gravity; $\mu_{\text{1}}$ and $\mu_{\text{2}}$ are the flow velocities at various points of the pump unit’s inlet and outlet flow passage cross-sections, respectively; Z_1_ and Z_2_ are the positional heads; and *ρ* is the fluid density.

In pump design, it is commonly assumed that the incoming flow at the inlet is irrotational and uniformly distributed. Consequently, a more uniform and perpendicular inflow is more conducive to improving operational efficiency. Flow velocity uniformity reflects the degree of flow velocity homogeneity at the outlet cross-section of the flow passage. It directly influences the suction conditions of the subsequent impeller and the energy conversion efficiency. A higher value indicates a more uniform velocity distribution. The flow velocity uniformity is defined as:


$$\begin{array}{c} V_{u}=\left(\text{1}-\frac{\text{1}}{{\overset{―}{V}}_{a}}\sqrt{\frac{\sum_{i=\text{1}}^{n}{\left(V_{ai}-{\overset{―}{V}}_{a}\right)}^{\text{2}}}{n}}\right)\times \text{1}\text{0}\text{0}\% \end{array}$$ (2)

The weighted average angle is defined as:


$$\begin{array}{c} \theta =\sum i=\text{1}n\frac{V_{ai}\left({\text{9}\text{0}}^{\circ }-{\text{tg}}^{-\text{1}}\frac{V_{ti}}{V_{ai}}\right)}{\sum_{i=\text{1}}^{n}V_{ai}} \end{array}$$ (3)

where $V_{u}$ is the axial velocity distribution uniformity at the outlet cross-section; ${\overset{―}{V}}_{a}$ is the average axial velocity on the outlet cross-section, in m/s; $V_{ai}$ is the axial velocity of computation cell ii on the outlet cross-section, in m/s; $V_{\text{ti}}$ is the tangential velocity of computation cell ii on the outlet cross-section, in m/s; *n* is the number of computation cells on the outlet cross-section; *θ* is the weighted average angle of the outflow velocity, in °.

The aforementioned metrics macroscopically define the evaluation criteria for the performance of the inflow and outflow passages. To further elucidate the mechanisms behind performance variations and identify locations of energy loss, the entropy production principle is employed for a more detailed analysis of the internal flow field. The entropy increase principle is one expression of the Second Law of Thermodynamics, stating that the infinitesimal increase in entropy is always greater than zero in an irreversible process. Entropy production refers to the inevitable loss caused by irreversibility during work transfer, whereby lost mechanical energy is converted into internal energy, resulting in energy dissipation. For turbulent flow, the entropy production rate can be divided into two components: one is the direct dissipation term due to mean velocity, termed direct entropy production; the other is the turbulent dissipation term due to fluctuating velocity, termed indirect entropy production, defined as:


$$\begin{array}{c} {\dot{S^{\prime \prime \prime }}}_{D}={\dot{S^{\prime \prime \prime }}}_{\overset{―}{D}}+{\dot{S^{\prime \prime \prime }}}_{D^{\prime }} \end{array}$$ (4)

where ${\dot{S^{\prime \prime \prime }}}_{D}$ is the total entropy production rate, in W/(m^3^·K); ${\dot{S^{\prime \prime \prime }}}_{\overset{―}{D}}$ is the entropy production rate caused by the mean velocity; ${\dot{S^{\prime \prime \prime }}}_{D^{\prime }}$ is the entropy production rate caused by the fluctuating velocity. The entropy production due to the mean velocity is given by:


$$\begin{array}{c} \begin{array}{c} {\dot{S^{\prime \prime \prime }}}_{\overset{―}{D}}\text{=}\frac{\mu }{T}\left[{\left(\frac{\partial \overset{―}{u}}{\partial y}+\frac{\partial \overset{―}{v}}{\partial x}\right)}^{\text{2}}+{\left(\frac{\partial \overset{―}{u}}{\partial z}+\frac{\partial \overset{―}{w}}{\partial x}\right)}^{\text{2}}+{\left(\frac{\partial \overset{―}{v}}{\partial z}+\frac{\partial \overset{―}{w}}{\partial y}\right)}^{\text{2}}\right]\\ \text{\textbackslash{}hspace\{1em}\}+2\frac{\mu }{T}\left[{\left(\frac{\partial \overset{―}{u}}{\partial x}\right)}^{\text{2}}+{\left(\frac{\partial \overset{―}{v}}{\partial y}\right)}^{\text{2}}+{\left(\frac{\partial \overset{―}{w}}{\partial z}\right)}^{\text{2}}\right] \end{array} \end{array}$$ (5)

The entropy production due to the fluctuating velocity is given by:


$$\begin{array}{c} \begin{array}{c} {\dot{S^{\prime \prime \prime }}}_{D^{\prime }}\text{=}\frac{\mu_{eff}}{T}\left[{\left(\frac{\partial u^{\prime }}{\partial y}+\frac{\partial v^{\prime }}{\partial x}\right)}^{\text{2}}+{\left(\frac{\partial u^{\prime }}{\partial z}+\frac{\partial w^{\prime }}{\partial x}\right)}^{\text{2}}+{\left(\frac{\partial v^{\prime }}{\partial z}+\frac{\partial w^{\prime }}{\partial y}\right)}^{\text{2}}\right]\mathrm{\backslash hfill}\\ +2\frac{\mu_{eff}}{T}\left[{\left(\frac{\partial u^{\prime }}{\partial x}\right)}^{\text{2}}+{\left(\frac{\partial v^{\prime }}{\partial y}\right)}^{\text{2}}+{\left(\frac{\partial w^{\prime }}{\partial z}\right)}^{\text{2}}\right]\mathrm{\backslash hfill} \end{array} \end{array}$$ (6)

where $\overset{―}{u}$, $\overset{―}{v}$, and $\overset{―}{w}$ are the components of the mean velocity in the x, y, and z directions, respectively; $u^{\prime }$, $v^{\prime }$, and $w^{\prime }$ are the corresponding fluctuating velocity components in the x, y, and z directions; μ is the dynamic viscosity of the fluid, in Pa·s; $\mu_{eff}$ is the effective viscosity of the fluid; and *T* is the temperature. In the present CFD simulations, the temperature *T* is treated as a constant (298.15 K) because the flow in the pump unit is essentially isothermal. The temperature rise due to viscous dissipation is negligible for water pumps under normal operating conditions, and thus the assumption of constant temperature does not introduce appreciable error in entropy production estimation.

## 3. CFD-based numerical simulation and optimization of the vertical mixed-flow pump system

### 3.1 CFD optimization design of the inlet flow passage

#### 3.1.1 Parameter design of the inlet flow passage.

**Fig 2:**
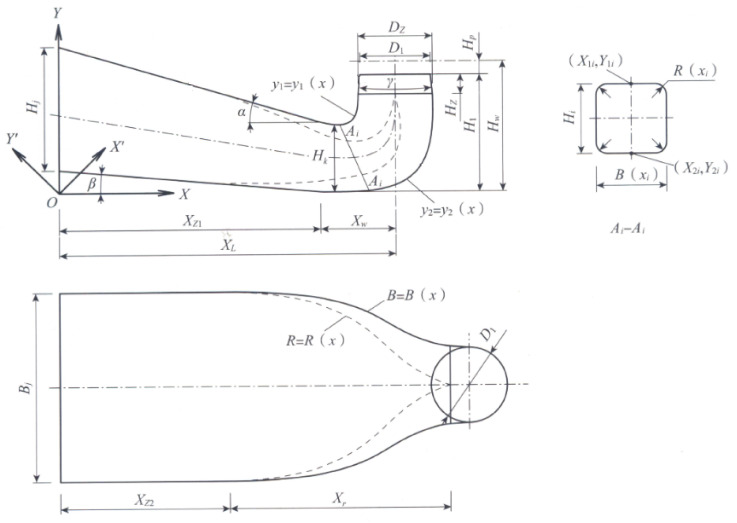
Schematic diagram of single-line layout for elbow-type inlet flow passage.

The designed elbow-type inlet flow passage consists of a straight section, a curved section, and an outlet conical section, with its main structure and dimensions illustrated in Fig 2. The profile control dimensions include the width *B*_*j*_, length *X*_*L*_, height *H*_*w*_, throat height *H*_*k*_, straight-section length *Xz*_*1*_, curved-section length *X*_*w*_, transition arc length *X*_*r*_ inlet height *H*_*j*_, upper-plate inclination angle α, and bottom-plate inclination angle *β*. Based on statistical data from existing large-scale elbow-type pumping stations in China, the reasonable ranges for the core control dimensions were determined as follows: the width of the elbow passage *B*_*j*_=(2.0 ~ 2.5)*D* (where DD is the impeller diameter, and the maximum value can reach 2.8DD), the length *X*_*L*_=(3.5 ~ 4.0)*D*(the length also needs to comprehensively consider requirements such as the layout of the powerhouse and the service bridge), the height *H*_*w*_=(1.8 ~ 1.9)*D*, the throat height *H*_*k*_=(0.8 ~ 1.1)*D*, the curved-section length *X*_*w*_=(1.0 ~ 1.1)*D*, and the transition arc length *X*_*r*_=(2.2 ~ 2.5)*D*. To justify this selection, a preliminary sensitivity analysis was conducted based on previous studies on elbow-type intake passages. According to the literature [6,7,38], parameters such as the upper‑plate inclination angle *α* and bottom‑plate inclination angle *β* have relatively minor effects on hydraulic performance compared to throat height, curved‑section length, and transition arc length, as their variation ranges are constrained by the inlet height and forebay bottom elevation. Specifically, *α* is determined by *H*_*j*_, *H*_*k*_, and *X*_*w*_, and typically varies within 12°–30°, while β is less than 10° and its influence on flow uniformity is secondary. Therefore, the three selected parameters are the most sensitive and were prioritized for orthogonal optimization. The upper-plate inclination angle αα is determined by the inlet height *H*_*j*_, throat height *H*_*k*_, and curved-section length *X*_*w*_, generally ranging from 12∘to 30∘. The bottom-plate inclination angle ββ is determined by the flow passage height *H*_*w*_ and the forebay bottom elevation, with *β* < 10°. The inlet height *H*_*j*_ was designed to ensure an average inlet velocity *V*_in_<1.0m/s under the design flow rate while maintaining sufficient submergence depth at the flow passage entrance.

Based on the core layout constraints of the pumping station powerhouse, the inlet width of the inlet flow passage was fixed at 5.80 m, and the horizontal distance from the inlet section to the impeller center was set at 7.94 m. Guided by engineering practice and literature review, three key parameters with the highest sensitivity to hydraulic performance were selected as the factors for the orthogonal test. The study focused primarily on the parametric variations of the throat height, the curved-section length, and the transition arc length. Three levels were assigned to each factor, covering the core recommended values within the aforementioned reasonable ranges, as detailed in Table 2.

**Table 2:** Core parameters of inlet flow passage optimization schemes.

Factor	Symbol	Level 1	Level 2	Level 3
Throat Height	A	0.90 *D*	1.00 *D*	1.10 *D*
Curved-section Length	B	1.00 *D*	1.05 *D*	1.10 *D*
Transition Arc Length	C	2.30 *D*	2.40 *D*	2.50 *D*

Nine sets of experiments, designated as JS1 through JS9, were designed using an L9(3^3^) orthogonal array to comprehensively cover all combination scenarios of the three parameters. The experimental scheme and parameter combinations are detailed in Table 3.

**Table 3:** Orthogonal experimental design matrix (L9(3^3^)) for inlet flow passage.

Case	Throat Height A	Curved-section Length B	Transition Arc Length C
JS1	0.90D (Level 1)	1.00D (Level 1)	2.30D (Level 1)
JS2	0.90D (Level 1)	1.05D (Level 2)	2.40D (Level 2)
JS3	0.90D (Level 1)	1.10D (Level 3)	2.50D (Level 3)
JS4	1.00D (Level 2)	1.00D (Level 1)	2.40D (Level 2)
JS5	1.00D (Level 2)	1.05D (Level 2)	2.50D (Level 3)
JS6	1.00D (Level 2)	1.10D (Level 3)	2.30D (Level 1)
JS7	1.10D (Level 3)	1.00D (Level 1)	2.50D (Level 3)
JS8	1.10D (Level 3)	1.05D (Level 2)	2.30D (Level 1)
JS9	1.10D (Level 3)	1.10D (Level 3)	2.40D (Level 2)

**Fig 3:**
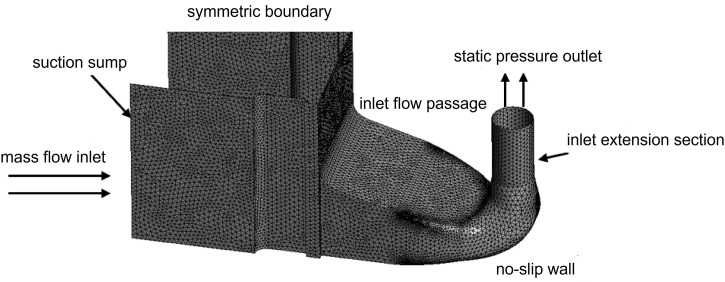
Computational domain and mesh generation of the inlet flow passage for vertical mixed-flow pump unit.

The computational domain for the numerical simulation of the inlet flow passage included a portion of the forebay, the inlet flow passage itself, and an extension section, which was discretized using an unstructured mesh. The mesh for the inlet flow passage comprised approximately 1.21 million cells. A mesh independence check confirmed that this resolution provided an optimal balance between computational accuracy and efficiency. Specifically, three mesh densities (0.86 million, 1.21 million, and 1.58 million cells) were compared under the design flow condition. The variation in hydraulic loss between the 1.21‑million and 1.58‑million cell meshes was less than 0.5%, while the 0.86‑million cell mesh yielded a deviation of 3.2% relative to the finest mesh. Therefore, the 1.21‑million cell mesh was adopted for all subsequent simulations to balance accuracy and computational cost. All CFD simulations were performed using ANSYS Fluent. The realizable k‑ε turbulence model with scalable wall functions was employed, as it has been widely validated for pump flow simulations [19]. The convergence criterion was set to 1 × 10^−5^ for all residuals, and mass flow rate imbalance at the inlet and outlet boundaries was monitored to be less than 0.1%. Furthermore, the mesh distortion rate was maintained below 5%, meeting the requirements for reliable numerical simulation. Taking the inlet flow passage design JS1 as an example, the computational domain and the corresponding mesh for the vertical mixed-flow pump system are presented in Fig 3.

#### 3.1.2 Analysis of optimization results.

Based on the design discharge of the pumping station at 10.6 m^3^/s, a comparative analysis of the hydraulic performance of the nine design schemes was conducted, with the results presented in Table 4.

**Table 4:** Hydraulic performance of each inlet flow passage scheme.

Case	Hydraulic Loss (m)	Flow Velocity Uniformity (%)	Weighted Average Angle (°)
JS1	0.104	97.06	84.82
JS2	0.105	97.07	84.81
JS3	0.109	97.05	84.81
JS4	0.106	97.05	84.78
JS5	0.106	96.98	84.80
JS6	0.105	97.04	84.79
JS7	0.112	97.08	84.78
JS8	0.108	97.04	84.56
JS9	0.106	97.05	84.78

Range analysis was performed on the results of the nine experimental cases to quantify the influence intensity of each factor on the three evaluation metrics.

As shown in Table 5, the ranking of factors by their influence on hydraulic loss was: transition arc length (C)> throat height (A)> curved-section length (B). The transition arc length exhibited the largest range of 0.004, identifying it as the dominant factor. Level 2 corresponded to the minimum hydraulic loss of 0.106 m, while Level 3 resulted in the maximum loss of 0.110 m, indicating that an excessively long transition arc significantly increases energy dissipation. For throat height, Level 2 yielded the optimal hydraulic loss of 0.106 m, with Level 3 producing the greatest loss, which is attributed to degraded flow passage cross-section adaptability caused by an overly large throat height. The curved-section length had the smallest range of 0.001, rendering its influence on hydraulic loss negligible.

**Table 5:** Range analysis table of hydraulic loss.

Factor	Level 1	Level 2	Level 3	Range	Influence Ranking
A Throat Height	0.106	0.107	0.109	0.003	2
B Curved-section Length	0.107	0.107	0.108	0.001	3
C Transition Arc Length	0.106	0.106	0.110	0.004	1

As shown in Table 6, the ranking of factors by their influence intensity was: throat height (A)> curved-section length (B)> transition arc length (C). Throat height was identified as the core influencing factor, with Level 1 yielding the highest flow velocity uniformity of 97.06% and Level 2 the lowest at 97.06%, indicating that the extreme levels were more conducive to uniform velocity distribution. The curved-section length achieved its optimal uniformity of 97.06% at Level 1, though the differences from other levels were marginal. For the transition arc length, the variation in uniformity across all levels was ≤ 0.02%, demonstrating the weakest influence.

**Table 6:** Range analysis table of flow velocity uniformity.

Factor	Level 1	Level 2	Level 3	Range	Influence Ranking
A Throat Height	97.06	97.02	97.04	0.04	1
B Curved-section Length	97.06	97.03	97.03	0.03	2
C Transition Arc Length	97.04	97.05	97.03	0.02	3

As shown in Table 7, the weighted average angle was evaluated based on the absolute deviation Δθ from the ideal vertical inflow angle of 90°, where a smaller deviation indicates better performance. The ranking of factors by their influence intensity was: throat height (A)> transition arc length (C)> curved-section length (B). Throat height exhibited the most significant influence, with the smallest deviation of 5.19° at Level 1 and the largest deviation of 5.29° at Level 3, indicating that a smaller throat height brings the flow closer to the ideal vertical inflow. For the transition arc length, Level 3 yielded the optimal deviation of 5.20°, while Level 1 resulted in the poorest performance at 5.28°. The differences among the levels of the curved-section length were relatively small, demonstrating a limited influence.

**Table 7:** Range analysis table of deviation value *Δθ* of weighted average angle.

Factor	Level 1	Level 2	Level 3	Range	Influence Ranking
A Throat Height	5.19°	5.21°	5.29°	0.10°	1
B Curved-section Length	5.21°	5.28°	5.21°	0.07°	3
C Transition Arc Length	5.28°	5.21°	5.20°	0.08°	2

Based on the comprehensive analysis above, the transition arc length primarily governs hydraulic loss, while the throat height dominates both flow velocity uniformity and the weighted average angle. The influence of the curved-section length is negligible. The parameter combination of Design JS1, specifically a throat height of 0.90D, a curved-section length of 1.00D, and a transition arc length of 2.30D, aligns with the favorable levels for each individual performance indicator. Furthermore, the quantitative data presented in Table 8 confirm that JS1 exhibits the optimal comprehensive performance. Its hydraulic loss was only 0.104 m, representing a 7.1% reduction compared to the worst-performing design, JS7. The flow velocity uniformity reached 97.06%, merely 0.02% lower than the best-case design (JS7), a difference considered negligible in engineering practice. JS1 also achieved the smallest deviation from the ideal vertical inflow, with a weighted average angle offset of 5.18°, thereby effectively minimizing impact losses as the flow enters the impeller and enhancing energy efficiency.

**Table 8:** Comprehensive performance comparison of representative inlet flow passage schemes.

Performance Metric	Optimal Case	Metric Value	Second-best Case	Metric Value
Minimum Hydraulic Loss	JS1	0.104m	JS2、JS6	0.105m
Maximum Flow Velocity Uniformity	JS7	97.08%	JS2	97.07%
Minimum Weighted Average Angle Offset	JS1	5.18°	JS2	5.19°

To further evaluate the statistical significance of each factor’s effect, an analysis of variance (ANOVA) was performed for the hydraulic loss metric. The results show that the transition arc length (C) has the highest F‑value and a p‑value < 0.05, indicating a statistically significant effect on hydraulic loss. Throat height (A) also shows moderate significance (*p* < 0.10), while curved‑section length (B) is not statistically significant (*p* > 0.10). This confirms the range analysis ranking and provides quantitative confidence in the factor prioritization. Detailed ANOVA results are available from the corresponding author upon request.

To select the optimal scheme from the nine orthogonal cases, a multi‑criteria decision approach was adopted. Hydraulic loss was considered the primary indicator because it directly reflects energy efficiency. Among all schemes, JS1 exhibited the lowest hydraulic loss (0.104 m). Its flow velocity uniformity (97.06%) was only 0.02% lower than the maximum (97.08% of JS7), a difference well within the engineering tolerance. Its weighted average angle offset (5.18°) was the smallest, indicating the most vertical inflow. Therefore, JS1 was unambiguously selected as the optimal scheme without requiring additional weighting coefficients. For completeness, an entropy weight method was also applied post hoc, which consistently ranked JS1 as the best.

**Fig 4:**
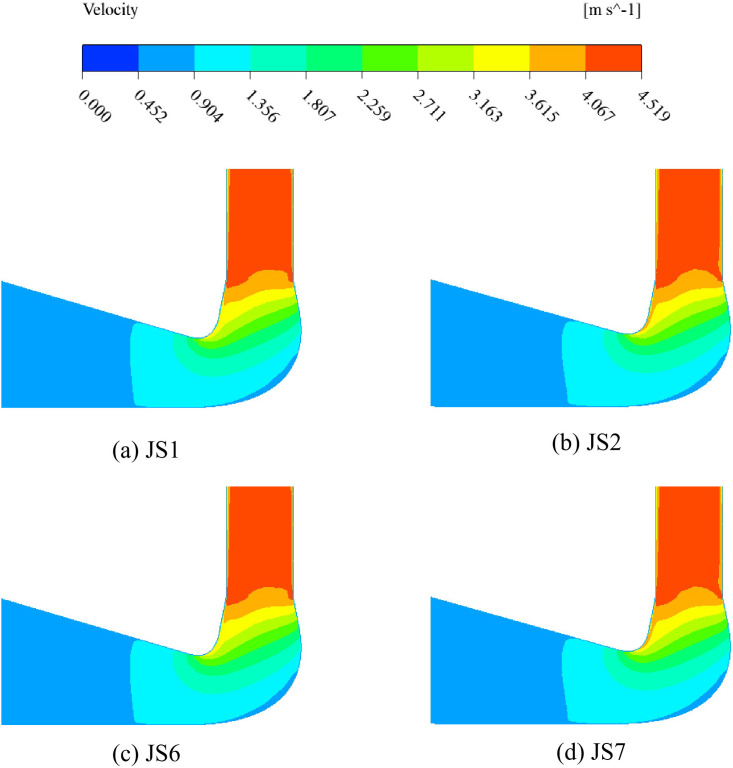
Contours of velocity distribution for typical inlet flow passage schemes (design flow rate). **(a)** JS1 scheme **(b)** JS2 scheme **(c)** JS6 scheme **(d)** JS7 scheme.

**Fig 5:**
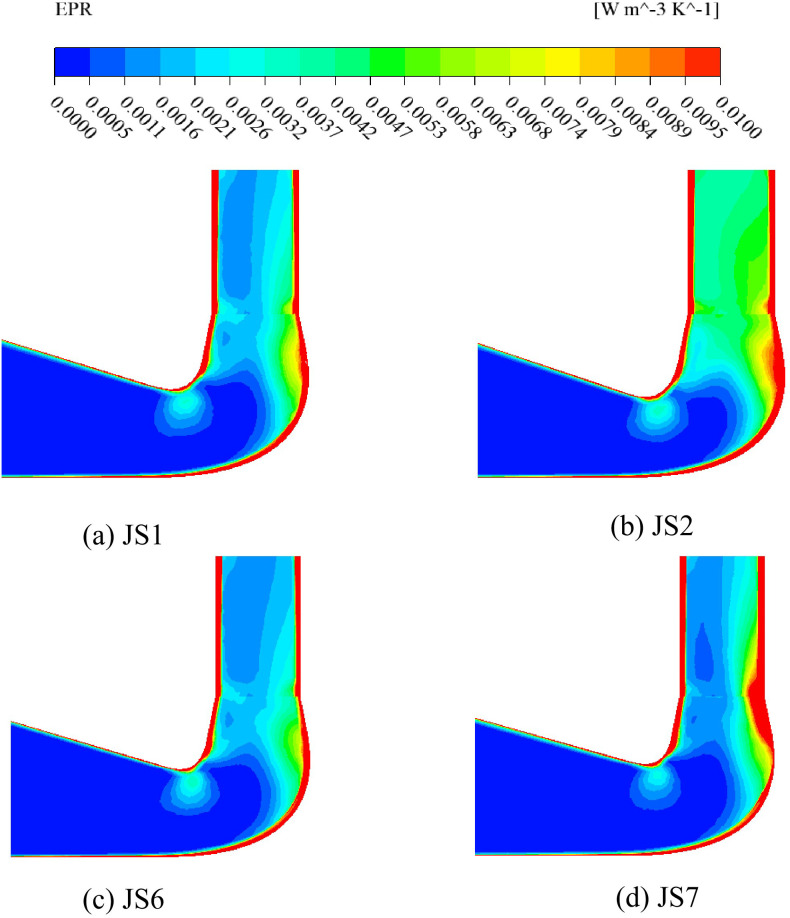
Contours of entropy production rate distribution for typical inlet flow passage schemes (design flow rate). **(a)** JS1 scheme **(b)** JS2 scheme **(c)** JS6 scheme **(d)** JS7 scheme.

To further validate the flow field advantages of the optimal design, four representative high-performance cases were selected for comparative flow field analysis, encompassing JS1 (minimum hydraulic loss), JS7 (maximum flow velocity uniformity), JS2 (second-best weighted average angle), and JS6 (second-best hydraulic loss). The results are presented in Figs 4 and 5. In designs JS2 and JS6, a localized high-velocity region with a maximum velocity of 3.6 m/s was observed along the inner side of the elbow section. The corresponding entropy production rate in this area exceeded 0.006 W/(m^3^·K), indicating a potential risk of flow separation. For design JS7, due to its throat height approaching the lower limit of the recommended range, velocity distortion occurred in the outlet section, forming a localized low-velocity zone. In contrast, design JS1 exhibited a gentle velocity gradient within the elbow section, with a maximum velocity of only 2.8 m/s. The entropy production was distributed uniformly, and the streamlines were smooth without recirculation. The flow accelerated through the straight section and turned smoothly in the curved section before entering the impeller chamber in a well-organized manner. The comprehensive flow field performance of JS1 was significantly superior to that of the other schemes.

### 3.2 CFD optimization design of the outlet flow passage

#### 3.2.1 Design of the outlet flow passage optimization scheme.

**Fig 6:**
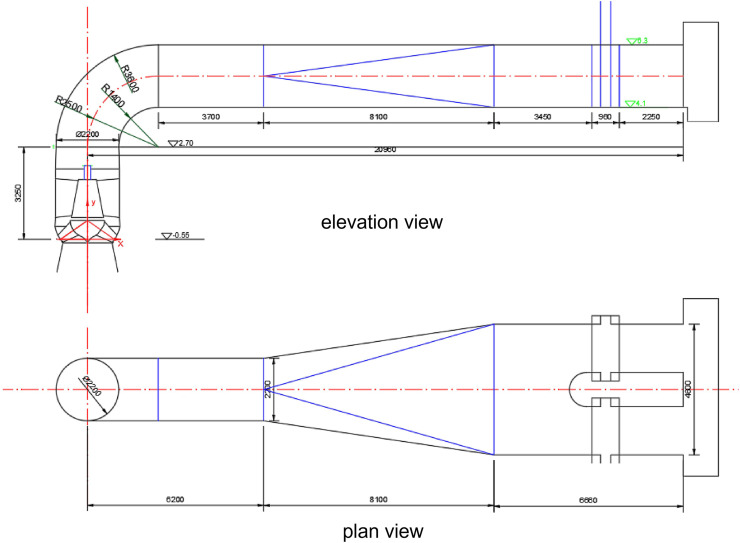
Schematic diagram of outlet flow passage design.

According to pump station design codes or specifications, the equivalent diffusion angle of the diffusion section in a straight pipe-type outlet flow passage must be controlled within a reasonable range. An excessively large angle can easily induce flow disorder, with the typical recommended range being 8° to 12°. The designed straight pipe-type outlet flow passage in this study strictly adhered to this specification, incorporating the equivalent diffusion angle of the diffusion section into the aforementioned reasonable interval. Its specific structural configuration, key dimensions, and layout are illustrated in Fig 6.

Considering the engineering constraints of the pumping station’s outlet flow passage, with the outlet section top elevation at ▽. 6.3 m, bottom elevation at ▽. 4.1 m, and an outlet width of 4.6 m, three outlet flow passage schemes, designated C1 to C3, were designed as shown in Table 9. The core difference among these schemes lies in their equivalent diffusion angle, aimed at analyzing the influence of this parameter on both hydraulic loss and engineering layout.

**Table 9:** Core parameters of outlet flow passage schemes.

Scheme	Equivalent Diffusion Angle (°)
C1	8.82
C2	8.56
C3	8.68

The numerical computational domain for the outlet flow passage included the 90° elbow, the straight pipe-type outlet flow passage, the horizontal straight pipe, and the outlet extension section. This domain was discretized using an unstructured mesh, comprising approximately 2.13 million cells. A mesh independence check verified that this mesh density provided an optimal balance between computational accuracy and efficiency. Furthermore, the mesh distortion rate was maintained below 5%, meeting the requirements for reliable numerical simulation.

**Fig 7:**
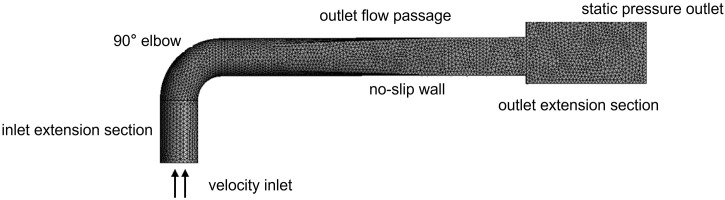
Computational domain and mesh generation of the outlet flow passage for vertical mixed-flow pump unit.

Since the inlet section of the outlet flow passage connects to the outlet section of the pump guide vane body—where the flow distribution is non-uniform—the computational domain was extended 1.5 times the impeller diameter upstream from the flow passage inlet section along its normal direction to accurately define the inlet boundary condition. The inlet boundary for the flow field computation was set at the inlet of this extended straight pipe. Taking the outlet flow passage scheme C1 as an example, its computational domain and mesh are presented in Fig 7.

#### 3.2.2 Analysis of optimization results.

Based on the design discharge of 10.6 m^3^/s, the three schemes were compared and the optimal one was comprehensively selected from the two dimensions of hydraulic loss and engineering feasibility. The hydraulic loss results presented in Table 10 show that Scheme C3 exhibited the minimum hydraulic loss, followed by Scheme C2, while Scheme C1 resulted in the maximum loss.

**Table 10:** Calculation results of hydraulic loss of outlet flow passage.

Scheme	Hydraulic Loss Δh (m)
C1	0.267
C2	0.239
C3	0.234

From an engineering implementation perspective, Scheme C3 cannot be directly implemented due to its relatively larger equivalent diffusion angle, which results in a length of the diffusion section that exceeds the reserved space in the powerhouse and creates a structural conflict with the foundation of the service bridge. As illustrated in Fig 6, the total length of the diffusion section in Scheme C3 would reach 5.32 m, exceeding the maximum allowable length of 4.95 m imposed by the distance between the pump outlet and the downstream service bridge foundation. This excess length would require encroaching into the bridge support zone, which is structurally unacceptable. In contrast, Scheme C1 has a diffusion section length of 4.82 m, which fits entirely within the existing powerhouse layout without any interference. The 0.50 m difference in length directly determines the feasibility of implementation. For Scheme C2, the length of the diffusion section exceeded the reserved space in the powerhouse by 0.3 m, necessitating a re-layout of the pipeline and modifications to the foundation structure. This adjustment would incur an approximate 15% increase in additional engineering costs, rendering it economically unfavorable. In contrast, the diffusion section length of Scheme C1 fully conforms to the existing powerhouse layout, requiring no additional modifications and presenting low construction difficulty, thereby satisfying all requirements for practical engineering implementation. Based on the comprehensive analysis above, Scheme C1 is determined to be the optimal outlet flow passage design.

### 3.3 CFD Simulation of the overall pump unit performance

**Fig 8:**
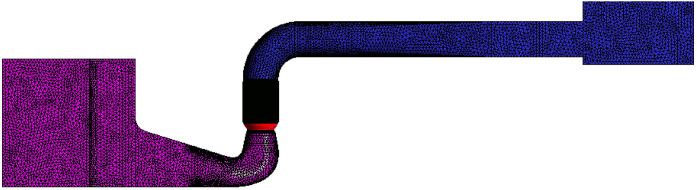
Computational domain of the pump unit.

The optimal inlet flow passage (JS1) and the optimal outlet flow passage (C1) were integrated with core flow-through components, including the mixed-flow pump impeller and guide vane body, to construct a complete pump unit for numerical simulation. The computational domain encompassed the forebay, inlet flow passage, pump section, and outlet flow passage. A reference coordinate system was established with the impeller center as the origin, as shown in Fig 8. The total mesh count was approximately 8 million cells. The inlet boundary condition was set as mass flow inlet corresponding to the design flow rate (10.6 m^3^/s), and the outlet boundary condition was set as pressure outlet (atmospheric pressure). No‑slip wall conditions were applied to all solid surfaces. The working fluid was water at 25°C with constant density (998.2 kg/m^3^) and dynamic viscosity (1.003 × 10^−3^ Pa·s).

The external characteristics and energy performance of the pump unit are presented in Table 11. The efficiency of the pump unit initially increased and then decreased with varying flow rates, reaching its peak at a flow rate of 11.66 m^3^/s. Under the design flow condition of 10.6 m^3^/s, the head was 7.59 m and the efficiency was 79.18%, achieving the design target.

**Table 11:** Energy characteristic table of prototype pump unit.

Flow Rate Q (m^3^/s)	Head H (m)	Efficiency η (%)
8.48	9.48	70.58
9.54	8.55	75.09
10.6	7.59	79.18
11.66	6.28	81.01
12.72	4.92	80.98
13.78	3.32	75.03
14.31	2.47	67.84

**Fig 9:**
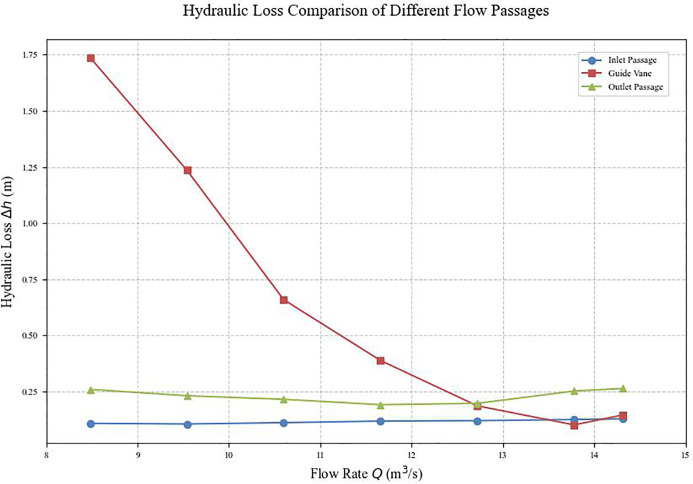
Hydraulic losses of flow-through components in the pump unit.

The distribution of hydraulic losses across the flow-through components of the pump unit under different flow conditions is shown in Fig 9. The relationship between the hydraulic loss of each component and the flow rate exhibited a parabolic curve (opening upward) across all flow conditions. Under low-flow conditions, a strong pre-rotation effect induced by the impeller near the outlet of the inlet flow passage led to an increase in the hydraulic loss of the inlet flow passage. As the flow rate increased, this pre-rotation effect weakened, and the hydraulic loss of the inlet flow passage showed an increasing trend with the flow rate. Near the optimal operating point of the pump unit, the matching between the guide vanes and the flow exiting the impeller was the best, resulting in relatively low internal hydraulic losses within both the guide vanes and the outlet flow passage. When operating away from the optimal point, the hydraulic losses in the guide vanes and the outlet flow passage tended to increase. An unexpected trend is observed in Fig 9 for the inlet flow passage: its hydraulic loss first decreases slightly from the lowest flow rate (8.48 m^3^/s) to near the design point (10.6 m^3^/s), and then increases with further flow rate increase. This non-monotonic behavior is attributed to the competing effects of pre-rotation and velocity magnitude. Under low-flow conditions, the impeller operates at a positive incidence angle, generating a strong pre-rotation vortex at the inlet passage outlet. This vortex induces additional mixing losses, elevating the hydraulic loss despite the low mean velocity. As the flow rate approaches the design point, the pre-rotation diminishes, and the loss drops to a minimum. Beyond the design point, the increase in mean flow velocity dominates, causing the loss to rise again. For the guide vanes and outlet passage, the loss is minimal near the design point due to optimal flow angle matching, and increases symmetrically on both sides. This trend is consistent with typical pump performance characteristics and further validates the accuracy of the numerical simulations. In the isolated flow passage simulation, the hydraulic loss for the JS1 inlet passage was 0.104 m, whereas in the integrated unit simulation, the loss for the same passage was 0.111 m, a difference of 0.007 m. This discrepancy primarily stems from the coupled flow field interactions—including the impeller’s pre-rotation effect and the influence of other components—present in the full-unit simulation, as opposed to the isolated flow field environment of the single-passage simulation. This observation is consistent with previous findings that the impeller-induced pre‑rotation and the downstream guide vane resistance can alter the inlet passage outlet boundary conditions, leading to a slight increase in hydraulic loss when simulated as part of the complete pump unit (see, e.g., Zhang et al. [6] and Wang et al. [7]). The error falls within the acceptable range for engineering applications, further validating the reliability and engineering applicability of the optimization results obtained from the single-passage simulations.

**Fig 10:**
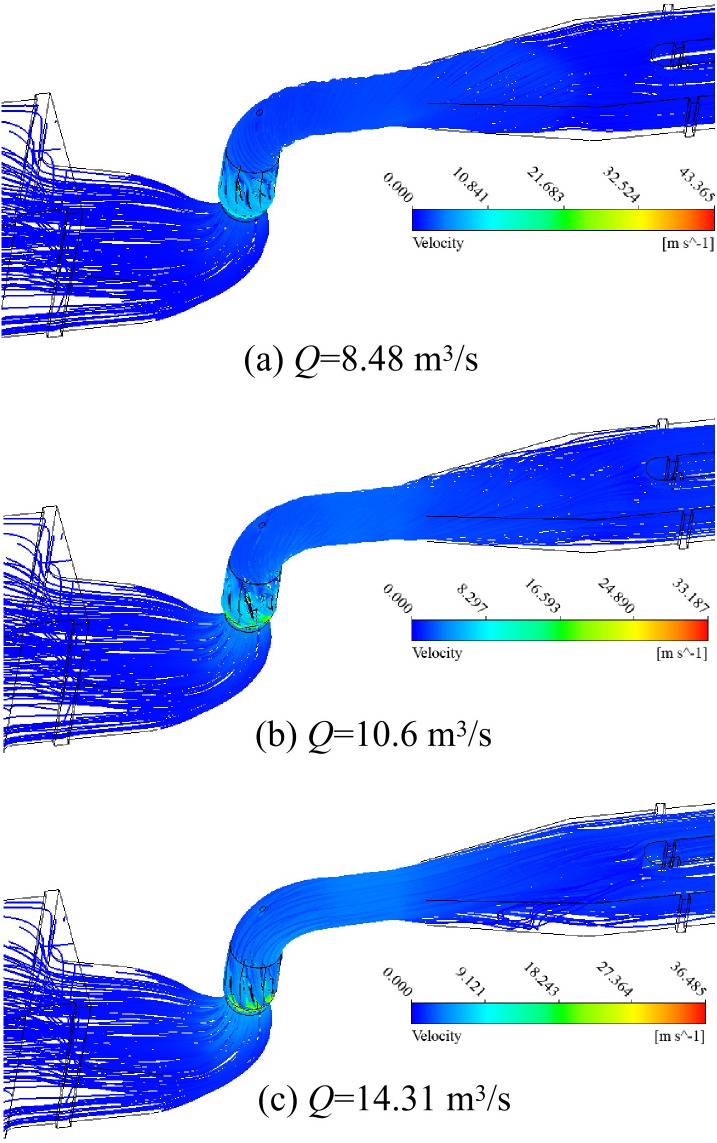
Internal streamlines of the prototype pump unit under different flow rate conditions.

To intuitively verify the overall flow field stability, tracer particles were uniformly distributed at the inlet of the pump unit flow passage to track their trajectories along the water flow. The internal flow field distribution of the pump unit was visually represented in the form of three-dimensional streamlines, as shown in Fig 10. Under different flow conditions, the streamlines within the inlet flow passage were smooth, without noticeable adverse flow patterns such as flow separation or vortices, indicating that the flow was steadily guided into the impeller. The streamlines in the outlet flow passage were well-organized, demonstrating sufficient conversion of kinetic energy to pressure energy, with no localized backflow or velocity distortion observed. The overall flow field exhibited excellent stability, which is consistent with the optimization results obtained from the individual flow passage simulations.

## 4. Model experimental study of the vertical mixed-flow pump unit

### 4.1 Model test methodology

**Fig 11:**
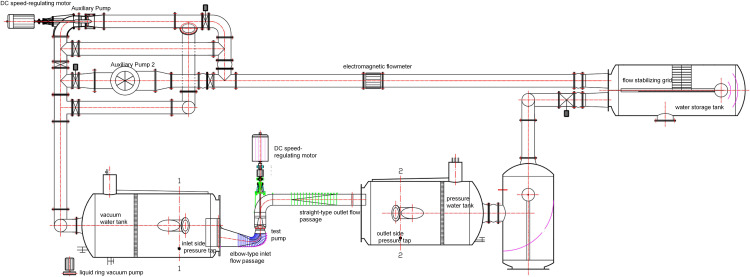
Schematic diagram of the high-precision pumping station test bench.

The tests were conducted on a high-precision pumping station test bench in a fluid power engineering laboratory, as shown in Fig 11. The test bench constituted a closed-loop system, equipped with hydraulic circulation, power, control, and measurement systems. The overall uncertainty for efficiency measurement was within ±0.288%, meeting the Grade 1 accuracy requirements of ISO 9906:2012, thus ensuring data reliability. The core measuring instruments are listed in Table 12.

**Table 12:** Parameters of main measuring equipment.

Measurement Item	Instrument Model	Range
Flow Rate	DN400 Electromagnetic Flowmeter	0 ~ 500 L/s
Head	EJA110A Differential Pressure Transmitter	0 ~ 250 kPa
Torque/Speed	JC1A Torque Meter	0 ~ 500 N·m
NPSH (Net Positive Suction Head)	EJA130 Absolute Pressure Transmitter	−100 ~ 100 kPa

The model pump unit tests strictly adhered to the Euler similarity criterion. The Reynolds number of the model pump unit under the design condition satisfied Re > 1 × 10^6^. The prototype impeller diameter for the pumping station was D = 1900 mm, and the model pump impeller diameter was D ~ m~ = 300 mm. Therefore, the geometric scale ratio of the model was:


$$\begin{array}{c} \lambda_{D}=\frac{D}{D_{\text{m}}}=\frac{1900}{300}=6.333 \end{array}$$ (7)

In addition to the Euler similarity, the model tests also satisfied the Reynolds number independence criterion. With *Re* > 1 × 10^6^ under the design condition, the flow is well into the fully turbulent regime, ensuring that viscous scale effects on the loss coefficients are negligible. The Froude number was not explicitly matched because free-surface effects are minimal in the confined intake and discharge passages, and the pump unit operates under pressurized flow conditions. Therefore, the Euler and Reynolds similarity criteria are considered sufficient for accurate performance prediction.

The prototype pump speed was *n* = 214.3r/min. Based on the relationship $nD=n_{m}D_{m}$, the rated speed for the model pump unit tests was calculated as follows:


$$\begin{array}{c} n_{\text{m}}=\frac{nD}{D_{\text{m}}}=\frac{214.3\times 1900}{300}=1357.2 \end{array}$$ (8)

During the tests, the operating speed of the model pump unit was adjusted to *n*_m_ = 1357.2r/min r/min via the DC motor controller and maintained constant throughout the energy performance test series to minimize interference from speed fluctuations on the experimental results.

### 3.2 Energy performance test

**Fig 12:**
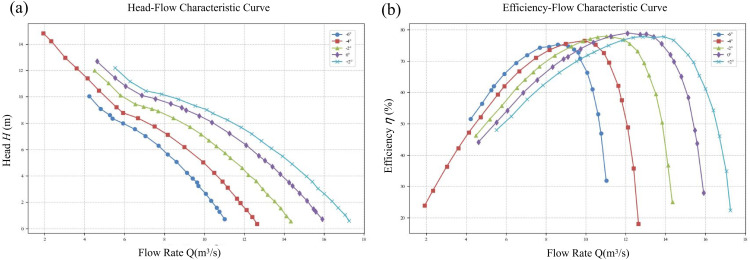
Energy characteristic curves of the model pump (n = 1357.2 r/min). (a) Q-H curve of the model pump (b) Q-η curve of the model pump.

The energy characteristic curves of the model pump are shown in Fig 12. At the design speed of 1357.2 r/min, the model pump achieved a flow rate of 283.7 L/s under the prototype head condition of 7.1 m, with an efficiency of 77.34%. The discrepancy between this experimental value and the CFD simulation result was 2.8%, which meets the required engineering accuracy. The peak efficiency point occurred at a flow rate of 301.5 L/s, with an efficiency of 78.98%. The high-efficiency range covered flow rates from 246.5 to 324.0 L/s, satisfying the operational requirement for a broad flow range.

**Fig 13:**
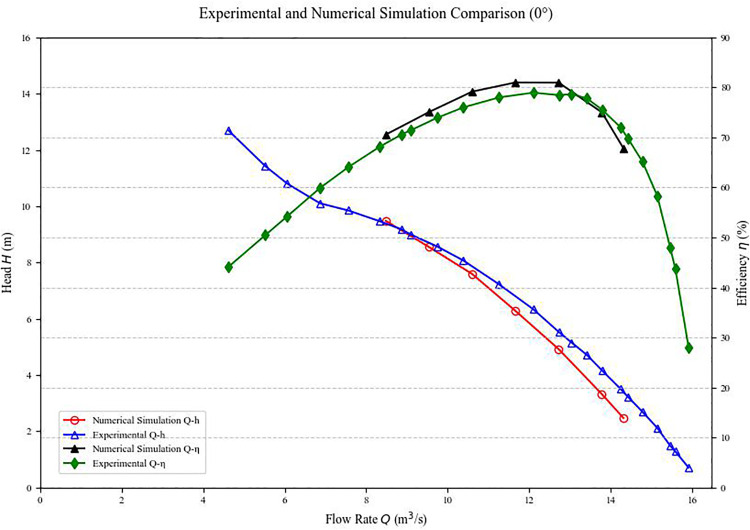
Comparison of pump external characteristics obtained by numerical calculation and experiment.

The positive head and efficiency obtained from the numerical calculations were slightly higher than the experimental values. Within the calculated flow rate range, the trends of the numerical results were in good agreement with the experimental data, as shown in Fig 13. Under the design flow condition, the errors for head and efficiency from the numerical simulation were less than 5%. This indicates that the numerical simulation possesses high predictive accuracy, and the numerical method is reasonable and reliable.

## 5. Conclusions

To address the influence of multi-structural parameter interactions in the inlet and outlet flow passages on the hydraulic performance of vertical mixed-flow pump units, a parameter weighting analysis method and structural optimization design based on orthogonal factors were proposed, significantly enhancing design efficiency. The main conclusions are as follows:

(1) The optimization methodology deeply coupling orthogonal experimental design with CFD efficiently overcomes the limitations of traditional single-factor optimization. Using an L9(3^3^) orthogonal array with throat height, curved-section length, and transition arc length as key factors, only nine simulation sets were required to cover full parameter combinations. This approach successfully quantified parameter interaction effects and their primary-secondary influences.
(2) The flow passage optimization yielded significant results. Through multi-scheme CFD optimization, the selected inlet flow passage JS1 achieved a hydraulic loss of 0.104 m and a flow velocity uniformity of 97.06%. The selected outlet flow passage C1 resulted in a hydraulic loss of 0.267 m, with no significant flow separation or recirculation observed within the passage. Subsequent model tests demonstrated that under the design head condition of 7.1 m, the prototype pump achieved a flow rate of 11.38 m^3^/s, an efficiency of 77.34%, and a shaft power of 958 kW. The performance of the pump unit fully meets the design requirements.
(3) Under the design condition, the discrepancies between the CFD simulations and the experimental external characteristics were within a reasonable range. This validates the accuracy of the CFD numerical simulations and confirms that the methodology can provide an effective technical reference for the efficient, stable, and economical optimization of large low-head pump units.
